# Glucagon-Like Peptide-1 Gene Therapy

**DOI:** 10.1155/2011/601047

**Published:** 2011-06-20

**Authors:** Anne M. Rowzee, Niamh X. Cawley, John A. Chiorini, Giovanni Di Pasquale

**Affiliations:** ^1^Molecular Physiology and Therapeutics Branch, National Institute of Dental and Craniofacial Research, Bethesda, MD 20892-2190, USA; ^2^Section on Cellular Neurobiology, Department of Health and Human Services, Eunice Kennedy Shriver National Institute of Child Health and Human Development, National Institutes of Health, Bethesda, MD 20892, USA

## Abstract

Glucagon-like peptide 1 (GLP-1) is a small peptide component of the prohormone, proglucagon, that is produced in the gut. Exendin-4, a GLP-1 receptor agonist originally isolated from the saliva of *H. suspectum* or Gila monster, is a peptide that shares sequence and functional homology with GLP-1. Both peptides have been demonstrated to stimulate insulin secretion, inhibit glucagon secretion, promote satiety and slow gastric emptying. As such, GLP-1 and Exendin-4 have become attractive pharmaceutical targets as an adjunctive therapy for individuals with type II diabetes mellitus, with several products currently available clinically. Herein we summarize the cell biology leading to GLP-1 production and secretion from intestinal L-cells and the endocrine functions of this peptide and Exendin-4 in humans. Additionally, gene therapeutic applications of GLP-1 and Exendin-4 are discussed with a focus on recent work using the salivary gland as a gene therapy target organ for the treatment of diabetes mellitus.

## 1. Introduction

A great deal of research has been performed on glucagon-like peptide-1 (GLP-1) since the initial characterization of glucagon-like substances secreted from the intestine in response to oral glucose [1]. A search of the literature identifies many articles describing original data as well as many excellent reviews (Holst [2], Riedel and Kieffer [3], and Brubaker [4]). Therefore, it is not the intent of this review to repeat what has previously been written, but to summarize what is known about GLP-1 with a focus on gene therapy using expression vectors of GLP-1 and other incretin mimetics in the salivary gland for the treatment of type 2 diabetes mellitus (T2DM).

## 2. Cell Biology

Glucagon-like peptide-1 (GLP-1(7–37)) is a 30 amino acid peptide that is initially synthesized as part of proglucagon, a prohormone composed of 180 amino acids (NCBI reference for human proglucagon, NP 002045.1). Within the proglucagon peptide lie the sequences of several smaller peptide hormones, such as glucagon, GLP-1, GLP-2, glicentin, and oxyntomodulin. Proglucagon is expressed in pancreatic **α**-cells within the Islets of Langerhans and also in intestinal endocrine L cells [5, 6]. In these specialized cells, proglucagon is intracellularly trafficked to the regulated secretory pathway where it can be processed into the smaller peptide hormone products indicated above. However, due to differential expression of the prohormone convertases (PCs) in each of these tissues [7–10], proglucagon is processed such that glucagon is produced in the pancreas by PC2 [11, 12] and GLP-1 is produced in the gut by PC1 [13–15]. The GLP-1 peptide can then undergo additional processing at both termini. At the amino terminus, six amino acids are removed to generate a new, mature N-terminus that is involved in activating the GLP-1 receptor in target tissues [16, 17]. The carboxyl terminus is trimmed by carboxypeptidase E [18] to remove two arginine residues, allowing a newly exposed glycine residue to be amidated by the enzyme, peptidyl alpha amidating mono-oxygenase [19]. GLP-1 is stored within secretory ranules of L cells until it is released in a stimulus dependent manner. GLP-1 is secreted into the bloodstream in response to taste receptor activation [20] and nutrients present in the digestive tract after a meal [21, 22] including glucose, amino acids, and, as recently demonstrated, some selected tetrapeptides [23]. In addition, neuroendocrine input [24] involving signaling by leptin, insulin, and gastric inhibitory peptide as well as muscarinic receptor activation [25] are involved in stimulated GLP-1 secretion from L cells.

## 3. Endocrinology

Following secretion of GLP-1 in vivo, it is estimated that approximately 30% of circulating GLP-1 survives long enough to reach the pancreas [26]. GLP-1 has a biological half-life of 2-3 min, similar to that of insulin, and is rapidly degraded by dipeptidyl aminopeptidase IV (DPP IV). Upon reaching the pancreas, GLP-1 induces the secretion of insulin from *β*-cells in a glucose-dependent manner as well as inhibits glucagon secretion from *α*-cells. Importantly, it has also been shown that GLP-1 enhances *β*-cell proliferation with a subsequent increase in *β*-cell mass (reviewed in [27]). This results in an increase in insulin availability under conditions of high demand and recovery of *β*-cell mass previously lost by a progressive reduction of pancreatic *β*-cell mass and function as a consequence of T2 DM. Additionally, GLP-1 restores glucose sensitivity to *β*-cells [28] and potentiates insulin-stimulated glucose utilization in pancreatized dogs [29]. GLP-1 also functions to slow gastric emptying [30], allowing a more controlled efflux of nutrients into the intestine and to the circulation. The controlled release of nutrients to the circulation in turn results in a more controlled nutrient uptake response by the tissues in the body. Additionally, a central role of GLP-1 in the regulation of satiety has been shown [31].

## 4. Exendin-4

Exendin-4 is a 39 amino acid peptide that shares similar functional properties with GLP-1 and was identified in the venom of the lizard, *Heloderma suspectum*, commonly known as the Gila monster (reviewed in [32]). While not a GLP-1 ortholog, exendin-4 shares 53% amino acid homology with full-length GLP-1. Due to a glycine residue in position 8, it is more resistant to degradation by DPP IV and hence has a longer biological half-life (~3-4 h) in the circulatory system [33]. As mentioned, exendin-4 acts as a GLP-1 receptor agonist and was shown to increase cAMP in guinea pig pancreas [34] and stimulate insulin secretion not only from mouse insulinoma cell lines but also rat Islet isolates [35]. Similar to that of GLP-1 in humans and mammals, exendin-4 has been shown to inhibit glucagon secretion, stimulate insulin secretion, protect against *β*-cell apoptosis, promote *β*-cell proliferation, promote satiety, and inhibit gastric emptying [32].

## 5. Gene Delivery of GLP-1 and Exendin-4 Constructs

The ability of GLP-1 and its analogues to stimulate insulin secretion in a glucose-dependent manner makes these molecules attractive potential therapies for T2 DM. However, the short half-life of GLP-1 in serum would necessitate repeated dosing, making injection with a recombinant peptide an expensive and inconvenient treatment strategy. To circumvent these shortcomings, many groups have explored alternative methods for expression and delivery of modified GLP-1 peptides and analogues including cell engineering [3, 36] and, more commonly, gene therapeutic approaches. 

Several groups have demonstrated expression and secretion of GLP-1 following delivery of viral or plasmid gene therapy vectors either via intravenous (IV) injection [37–42] or intraperitoneal injection [43, 44]. As a whole, these studies illustrate that in vivo expression of a transgenic GLP-1 peptide had positive effects on glucose homeostasis and could delay the onset of diabetes in both T1 and T2 DM models. Additionally, Samson and colleagues demonstrated that IV injection of a helper-dependent adenovirus encoding exendin-4 was able to improve glucose homeostasis in a T2 DM model [45]. While effective in animal models, the inherent risks involved in systemic administration of gene therapy vectors make targeted delivery of vectors an appealing alternative for treatment of patients.

Several studies from the laboratory of Dr. Q. Wang have used “plasmid-based, electroporation-enhanced intramuscular gene therapy” to target delivery of vectors encoding GLP-1 or exendin-4 fusion proteins into mouse models of DM [46–48]. In initial studies, an active GLP-1 peptide or an exendin-4 peptide was fused to the heavy chain constant regions (Fc regions) of mouse IgG to generate bivalent peptides that have putative increased half-life and potency in vivo. Plasmids encoding these constructs were directly injected into the muscle of mice with T1 DM (streptozotocin-induced) or T2 DM (db/db mice) and circulating fusion peptides were detected in plasma for at least 3-4 weeks following injection. The GLP-1 fusion peptide was shown to improve glucose tolerance in both models of DM [46, 47] and the exendin-4 fusion peptide was also shown to improve glucose homeostasis and ameliorate T1 DM symptoms when tested in mice treated with chronic low doses of streptozotocin [46]. 

The studies of the GLP-1 fusion peptide were taken a step further in 2010 when Liu and colleagues directed site-specific integration of the GLP-1/Fc fusion peptide plasmid DNA into the genome of transgenic mice carrying the human adeno-associated virus S1-(AAVS1-) integrating region [48]. Peak levels of circulating bivalent GLP-1 peptide were achieved 2 weeks following intramuscular injection and subsequent integration of the plasmid construct into genomic DNA, with sustained plasma levels through 10 weeks of analysis. When tested in high-fat-diet-challenged mice, those expressing the GLP-1 fusion peptide from muscle had decreased weight gain and food intake over time, retained normal levels of circulating leptin and ghrelin, and demonstrated an improved insulin response in an insulin tolerance test. When taken together, these studies demonstrate the potential of targeted gene therapy for treatment of diabetes and other endocrine deficiencies.

## 6. The Salivary Gland as a Gene Therapy Target Organ for the Treatment of T2 DM

The salivary gland (SG) has been demonstrated to be an effective target organ for gene therapy vectors in several animal models, and a Phase I/II clinical trial evaluating the safety and efficacy of recombinant adenoviral vector delivery to the parotid SG is currently ongoing [49]. Salivary glands possess many characteristics that make them suitable for gene transfer (reviewed in [50]). Perhaps foremost of these characteristics is that unlike liver or lung, SGs are not critical for life organs, and in the event of an adverse reaction a single SG may be removed with little effect on patient morbidity. Furthermore, because SGs are encapsulated, the likelihood of systemic viral vector spread and subsequent adverse reactions is much reduced. 

Salivary glands can produce and secrete large quantities of protein, an attribute making SGs highly suitable for gene therapy to treat single protein deficiency disorders. In addition to treating inducible models of endocrine disorders, it has recently been demonstrated that gene therapeutic vector delivery to SGs can be used to treat inborn genetic errors such as Fabry disease which exhibits a deficiency in the lysosomal enzyme alpha-galactosidase A [51]. Although SG epithelial cells are primarily exocrine, it is hypothesized that the SG constitutive secretory pathway (CSP) conveys transgenic proteins to the circulation via the basolateral membrane (reviewed in [49]). This has been supported by animal studies from our group demonstrating that therapeutically relevant levels of growth hormone and parathyroid hormone were present in serum following SG transduction, indicating the potential for SG gene therapy to treat endocrine disorders [52, 53]. 

As a proof-of-principle for SG-mediated treatment of diabetes, we have recently shown that transduction of mouse SGs with a recombinant adenovirus encoding a GLP-1 peptide is able to delay the onset of T1 DM in mice [54]. The adenovirus used in these studies (Ad-GLP-1) contains a modified human GLP-1 cDNA sequence encoding the active GLP-1(7–37) peptide with an Ala to Gly substitution at position eight to confer resistance to DPP-IV. This construct was demonstrated to produce a bioactive GLP-1 peptide that was resistant to DPP-IV degradation and was able to stimulate insulin secretion from pancreatic *β*-cells in vitro. 

Ad-GLP-1 was then delivered to the submandibular glands of intact mice by retroductal instillation [55] to determine the route of GLP-1 peptide secretion and the capacity for bioactivity in vivo. GLP-1 expressed by the SGs was detected in the serum of these mice and was able to rapidly reduce serum glucose levels in a glucose tolerance test when compared to animals treated with a control (Luciferase-containing, Ad-Luc) adenovirus. Furthermore, in keeping with the glucose-dependent mechanism of GLP-1 action, the blood glucose levels of fasted animals treated with Ad-GLP-1 were indistinguishable from those treated with Ad-Luc. 

After establishing that bioactive transgenic GLP-1 was secreted to the circulation, we tested the ability of SG-derived GLP-1 to ameliorate diabetes in an inducible model. In these studies, Ad-GLP-1 or Ad-Luc was delivered to SGs, one day later, all mice were then treated with the beta-cell-specific toxin alloxan, and blood glucose values of Ad-GLP-1- or Ad-Luc-treated mice were followed throughout the course of the experiment. As anticipated from previous experiments, GLP-1 expressed and secreted from SGs delayed the onset of alloxan-diabetes as demonstrated by significantly lower serum glucose levels than Ad-Luc-treated mice 48–72 h after alloxan treatment. We are currently generating adeno-associated viral (AAV) vectors capable of stable, long-term GLP-1 and exendin-4 expression in order to test the efficacy of SG-derived GLP-1 and its agonist in treating genetic models of T2 DM and obesity. Preliminary data strongly suggest that long-term expression may be achieved. 

Thus, the use of the SG as a tissue to express GLP-1 or exendin-4 by adeno- or adeno-associated viral vectors, demonstrate a means to accomplish a noninvasive delivery of gene therapy vectors for the treatment of DM.

## 7. Conclusion

The biology of GLP-1 and its potential use in gene therapy for the treatment of T2 DM is evident from the cache of papers available on the topic. GLP-1 appears to have all the requisite properties to maintain homeostatic levels of glucose in order to effectively treat T2 DM. Indeed, DPP IV inhibitors (e.g., Sitagliptin), which prolong the half-life of a patient's endogenous GLP-1, and synthetic GLP-1 receptor agonists (e.g., Exenatide and Liraglutide) are approved and on the market as adjunctive antidiabetic treatments. It is only a matter of time until a gene therapy approach appropriate for the clinic will develop and catch up to the small molecule industry of agonists, antagonists, and inhibitors. The first and most important barrier to cross in the transition from the laboratory to the clinic is demonstration of the safety of this approach. The use of the SG as a bioreactor represents one such tissue that would allow this question to be addressed with fewer complications, both physiologically and regulatory, compared to other potential gene therapy target tissues. It is apparent from the many successful attempts using a gene therapy approach to express GLP-1 or exendin-4 in animal models, that success is dependent on their sustained and, in the future, regulated expression.
